# Deletion of *Dtnbp1* in mice impairs threat memory consolidation and is associated with enhanced inhibitory drive in the amygdala

**DOI:** 10.1038/s41398-019-0465-y

**Published:** 2019-04-09

**Authors:** Cathy C. Y. Huang, Kevin J. Muszynski, Vadim Y. Bolshakov, Darrick T. Balu

**Affiliations:** 1 Department of Psychiatry, Harvard Medical School, Boston, MA USA; 2 Translational Psychiatry laboratory, McLean Hospital, Belmont, MA USA; 3 Cellular Neurobiology laboratory, McLean Hospital, Belmont, MA USA; 4 Present Address: Department of Life Sciences, National Central University, Taoyuan, Taiwan

## Abstract

Schizophrenia is a severe and highly heritable disorder. Dystrobrevin-binding protein 1 (*DTNBP1*), also known as dysbindin-1, has been implicated in the pathophysiology of schizophrenia. Specifically, dysbindin-1 mRNA and protein expression are decreased in the brains of subjects with this disorder. Mice lacking dysbinidn-1 also display behavioral phenotypes similar to those observed in schizophrenic patients. However, it remains unknown whether deletion of dysbindin-1 impacts functions of the amygdala, a brain region that is critical for emotional processing, which is disrupted in patients with schizophrenia. Here, we show that dysbindin-1 is expressed in both excitatory and inhibitory neurons of the basolateral amygdala (BLA). Deletion of dysbindin-1 in male mice (Dys^−/−^) impaired cued and context-dependent threat memory, without changes in measures of anxiety. The behavioral deficits observed in Dys^−/−^ mice were associated with perturbations in the BLA, including the enhancement of GABAergic inhibition of pyramidal neurons, increased numbers of parvalbumin interneurons, and morphological abnormalities of dendritic spines on pyramidal neurons. Our findings highlight an important role for dysbindin-1 in the regulation of amygdalar function and indicate that enhanced inhibition of BLA pyramidal neuron activity may contribute to the weakened threat memory expression observed in Dys^−/−^ mice.

## Introduction

Schizophrenia is a severe mental illness, characterized by positive (delusions), negative (anhedonia), and cognitive symptoms that affects 0.5–1% of population worldwide^1^. Dystrobrevin-binding protein 1 (*DTNBP1*), also known as dysbindin-1, has been implicated in the pathophysiology of schizophrenia. In human post mortem studies, both the mRNA and protein levels of dysbindin-1 were decreased in the dorsolateral prefrontal cortex (PFC) and hippocampus of subjects with schizophrenia^2–5^, brain regions that are critical for memory, cognition, and emotion. A recent study also demonstrated that human schizophrenia subjects with genetic variants in the gene that encodes dysbindin-1 (*DTNBP1*) exhibited cognitive dysfunction^6^. Moreover, dysbindin-1 knockout mice (Dys^−/−^) exhibit behavioral phenotypes similar to what is observed in schizophrenia, including reduced pre-pulse inhibition, impaired learning and memory, and decreased social interaction^7–13^. In sum, these data suggest that alterations in dysbindin-1 expression can contribute to the pathophysiology of schizophrenia.

Dysbinin-1 is a coiled-coil-containing protein that serves diverse functions, including synaptic homeostasis, exocytosis, synaptic vesicle biogenesis, and dendritic spine formation^14–18^. Dys^−/−^ mice exhibited impaired hippocampal long-term potentiation^19^, as well as disrupted excitatory and inhibitory synaptic transmission in the medial prefrontal cortex (mPFC)^9,20,21^. Additionally, abnormal dendritic morphology was found in hippocampal neurons in Dys^−/−^ mice in vitro^15,22^. These alterations observed in Dys^−/−^ mice demonstrate that dysbindin-1 may serve as a regulator of synaptic and structural plasticity.

The amygdala is located deep in the temporal lobe, consisting of many interconnected nuclei, and it plays key roles in emotional memory and processing of emotionally charged cues^23^. Clinical observations suggest that emotional perturbation is an important feature observed in patients with schizophrenia. Emerging evidence have further suggested that amygdalar dysfunction contributes to the pathophysiology of schizophrenia ^24–34^. Importantly, neuroimaging studies have shown that fear processing is impaired in patients with schizophrenia through inhibition of amygdala activity^33,35–37^. Thus, we hypothesized that dysbindin-1 may contribute to the regulation of threat memory-related behaviors by possibly regulating synaptic and neuronal functions in fear circuits in the brain, specifically in the amygdala, a key structure implicated in fear learning and memory^38^. In this study, we combined behavioral, electrophysiological, morphological and biochemical strategies, focusing on the analysis of Dys^−/−^ mice, to elucidate the functional roles for dysbindin-1 in neurotransmission in a fear-related neural circuit and in fear control.

## Material and methods

### Animals

Dys^−/−^ mice were a generous gift from Dr. Greg C. Carlson (University of Pennsylvania). Dys^−/−^ mice resulted from a spontaneous mutation on the DBA/2J background named Sandy^39^. Mice in this study were on a C57BL/B6J background, obtained by breeding the Dys^−/−^ mice on DBA/2J background with C57BL/B6J mice^40^. Heterozygous (Dys^+/−^) dysbindin-1 mice were backcrossed with C57BL/B6J mice to generate heterozygous dysbindin-1 breeders. Female and male Dys^+/−^ mice were bred to generate WT and Dys^−/−^ offspring. Adult male mice (3–5 months old) were used in this study. Animals were housed in groups of four at 22 °C under a 12:12-h light:dark cycle with lights on at 7:00A.M. Animals were provided with food and water ad libitum. Sandy Forward (5′-TCC TTG CTT CGT TCT CTG CT-3′), Sandy Reverse (5′-CTT GCC AGC CTT CGT ATT GT-3′) WT-SE3F (5′-TGA GCC ATT AGG AGA TAA GAG CA-3′), and WT-SE3R (5′-AGC TCC ACC TGC TGA ACA TT-3′) primers were used for the genotyping assays. All animal care and experimental procedures were approved by the McLean Hospital Institutional Animal Care and Use Committee.

### Immunoblot analysis

The amygdala samples containing the basolateral amygdala (BLA) and the basomedial amygdala were dissected out (bregma –1.2 to −2.3) as previously described^41^. SDS–PAGE and immunoblotting using brain tissue samples were performed and analyzed as previously described^42,43^. The primary antibodies used in this study were: anti-β-actin (ab8227; Abcam), anti-Arc (sc-166461; Santa Cruz Biotechnology), anti-phospho-CaMKIIα (sc-12886; Santa Cruz Biotechnology), CaMKIIα (sc-13141; Santa Cruz Biotechnology), anti-dysbindin-1 (11132-1-AP; Proteintech), anti-PSD95 (51-6900; Thermo Fisher Scientific). The values obtained from Dys^−/−^ mice were subsequently normalized to the WT groups. Results are expressed as a percentage of WT.

### Synaptosomal plasma membrane preparation

Mice were decapitated and amygdala tissue from both hemispheres was dissected out on ice. One sample contained tissues from three mice which were pooled together. The pooled tissues were homogenized in 500 µl ice-cold sucrose buffer (0.32 M sucrose/4 mM HEPES, pH7.4) for amygdala (1000 µl for hippocampus) using a 3 ml tissue grinder for 20–30 strokes, and then centrifuged at 800 g at 4 °C for 10 min. The pellets were suspended with 200 µl of the same sucrose buffer for amygdala (400 µl for hippocampus) and centrifuged again at 800 g at 4 °C for 10 min in order to obtain more supernatant fraction. The combined supernatants (S1) were centrifuged at 12,000 g at 4 °C for 15 min. The crude synaptosomal fraction (P2) was suspended in 600 µl buffer (4 mM HEPES, pH 7.4; 1200 µl for hippocampus) and rotated at 4 °C for 1 h. The samples were then centrifuged at 21,130×*g* at 4 °C for 30 min. The pellets were sonicated with 50 µl buffer for amygdala (50 mM HEPES, pH 7.4, 2 mM EGTA; 100 µl for hippocampus) and stored at −80 °C until use. All buffers used for SPM preparation contained a cocktail of phosphatase and protease inhibitors and EDTA.

### Immunofluorescence

Dual antigen immunofluorescence was performed as previously described^41^. Brain sections were incubated with primary antibody overnight at 4 °C. The primary antibodies used in this study were: anti-dysbindin-1 (11132–1-AP; Proteintech), anti-neuronal nuclei (MAB377; Millipore). Sections were then incubated with goat-anti-rabbit Alexa 488 and goat-anti-mouse Alexa 555 accordingly for 2 h at room temperature, followed by incubation with the DNA-specific fluorescent probe (DAPI) or Hoechst for 10 min. Images were taken using a Leica SP8 confocal microscope or Zeiss Axio Imager.M2. All experiments were repeated three times using different mice. For cell counting, every 6th brain section was selected for analysis. We analyzed 4–5 brain sections per animal from a total of three animals.

### Immunohistochemistry

The procedure was performed as previously described^41^. Brain sections were incubated with anti-parvalbumin (PV, P3088; sigma) primary antibody overnight at 4 °C. Sections were then incubated with biotinylated anti-mouse (BA-2000; Vector laboratories) antibody for 2 h at room temperature, followed by streptavidin horseradish peroxidase (434323; Invitrogen) for 2 h at room temperature, before reaction with 3,3′-diaminobenzidine tetrahydrochloride (D5905; Sigma). For cell counting, every 6th brain section was selected. We analyzed 4–5 brain sections per animal from a total of four animals.

### Electrophysiology

Animals were sacrificed and 250-μm coronal sections containing the BLA were prepared using a vibratome (VT1000, Leica) in an ice-cold N-methyl-d-glucamine (NMDG)-based cutting solution. The NMDG-based cutting solution contained the following (in mmol/l): 2.5 KCl, 20 HEPES, 1.2 NaH_2_PO_4_, 93 NMDG, 30 NaHCO_3_, 25 glucose, 5 sodium ascorbate, 3 sodium pyruvate, 5 N-acetylcyctine, 0.5 CaCl_2_, 10 MgCl_2_, saturated with 95% O2 and 5% CO2 with an osmolarity of 300–305 mOsm. Slices were incubated in the same NMDG-based cutting solution for 10 min at 32 °C before transferring to HEPES solution (in mmol/l): 92 NaCl, 2.5 KCl, 1.2 NaH_2_PO_4_, 20 HEPES, 30 NaHCO_3_, 25 glucose, 2 CaCl_2_, 2 MgCl_2_, 5 sodium ascorbate, 3 sodium pyruvate, and X 5-acetylcyctine (300–305 mOsm) at 24 °C for at least 1 h, where they remained until being transferred to the recording chamber. The external solution for recording contained (in mmol/l): 113 NaCl, 2.5 KCl, 2.5 CaCl_2_, 1.2 MgCl_2_, 1 NaH_2_PO_4_, 26 NaHCO_3_, 1 sodium ascorbate, 3 sodium pyruvate, 20 glucose, saturated with 95% O_2_ and 5% CO_2_ (300–305 mOsm). Slices were maintained at 32 °C throughout all recordings.

Whole-cell patch-clamp recordings were obtained using a MultiClamp 700B (Molecular Devices) amplifier and Digidata 1440 A with Clampex10.6 software. Signals were sampled at 5 kHz and filtered at 1 kHz. Recordings were performed using glass microelectrodes (2–4 MΩ), which were pulled with a horizontal puller (P-97, Sutter Instruments). For voltage-clamp experiments, the pipette solution contained (in mmol/l): 119 CsMeSO4, 8 tetraethylammonium chloride, 15 *N*-2-hydroxyethylpiperazine-*N*-2-ethanesulfonic acid, 0.6 ethylene glycol bis-2-aminoethyl ether-*N*,*N*′,*N*′,*n*′-tetraacetic acid, 0.3 Na3GTP, 4 MgATP, 5 QX-314.Cl, and 7 Na_2_CrPO_4_ (pH 7.2–7.3) with an osmolality of 280 mOsm. Under voltage clamp recording conditions, pyramidal neurons in the amygdala were kept at holding potentials specified within the description of specific experiments. For current-clamp recording, the recording electrode contained (in mmol/l): 123 potassium gluconate, 10 *N*-2-hydroxyethylpiperazine-*N*-2-ethanesulfonic acid, 0.2 ethylene glycol bis-2-aminoethyl ether-*N*,*N*′,*N*″,*n*′-tetraacetic acid, 8 NaCl, 2 MgATP, 0.3 NaGTP (pH 7.2–7.3), with an osmolality of 270–280 mOsm. Rheobase currents were measured under the current-clamp mode by injection of a series of 500-ms steps at 10-pA increments. The rheobase current was defined as the first current step capable of inducing one action potential. The firing frequency of pyramidal neurons was calculated from the number of action potentials generated by 500-ms-long current injections ranging from 50 to 300 pA with 10-pA increments. The intrinsic membrane properties of BLA neurons were assessed without blockers of ion channels or receptor antagonists (neither glutamate or GABA receptors were blocked) in the external medium. To record miniature inhibitory postsynaptic currents (mIPSCs), the external solution contained TTX (1 μM), as well as 6,7-dinitroquinoxaline-2,3-dione (DNQX; 50 μM) and 2-amino-5-phosphonopentanoic acid (APV; 50 μM) to block sodium channels, α-amino-3-hydroxy-5-methyl-4-isoxazolepropionic acid receptors (AMPARs), and N-methyl-d-aspartate receptors (NMDARs), respectively.

To record electrical stimulation-induced EPSCs (eEPSC) or IPSCs (eIPSC), the stimulating electrode was positioned at the internal capsule (thalamic input^44^) and stimulation pulses were delivered at a 0.05 Hz frequency. To determine the AMPAR/NMDAR amplitude ratio, AMPAR EPSCs were recorded first at −70 mV, and NMDAR EPSCs were then recorded at +40 mV in the presence of the GABA_A_ receptor antagonist picrotoxin. To calculate the AMPAR/NMDAR EPSC amplitude ratio, the average peak amplitude of AMPAR EPSC traces during last 5 min of the recording (15 traces) at −70 mV were divided by the average amplitude of NMDAR-mediated component of the EPSC measured 40 ms after the peak at +40 mV. To obtain the paired-pulse ratio (PPR) estimates, we recorded evoked AMPAR EPSCs at −70 mV, triggered by paired pulses with a 50-ms inter-pulse interval. The PPR was calculated by dividing the second EPSC amplitude by the first EPSC amplitude. To obtain the IPSC/EPSC amplitude ratio values, we first recorded EPSCs at a holding potential of −70 mV under voltage-clamp condition. Then, IPSCs were recorded in the same neuron at a holding potential of 0 mV (as described in ref. ^45^), induced by presynaptic pulses of the same intensity as were used to trigger EPSCs. The holding potential of −70 mV is close to the reversal potential for GABA_A_ receptor-mediated IPSC, whereas 0 mV is close to the reversal potential for the AMPA receptor (AMPAR)-mediated EPSC. Therefore holding the recorded neuron at −70 or 0 mV sequentially, we are able to record the isolated AMPAR EPSC or GABA_A_ receptor-mediated IPSC, respectively. To calculate the IPSC/EPSC ratio, the average peak amplitude of IPSCs (15 traces) at 0 mV was divided by the average amplitude of AMPAR EPSCs at −70 mV.

### Dendritic morphological analyses

Animals were transcardially perfused with 4% paraformaldehyde/0.125% glutaraldehyde in 0.1 M phosphate buffer (pH 7.4). The BLA was visualized on a Zeiss Axio Examiner A.1 microscope under the guidance of DAPI. Pyramidal neurons in the BLA were iontophoretically microinjected with Lucifer Yellow dye using a DC current (1–10 nA) for 10 min (or until distal processes were filled with dye). Imaging procedures and analysis criteria for filled neurons were as previously described^46,47^. Raw z-stack images were deconvolved in AutoQuant (Media Cybernetics) and analyzed automatically using NeuronStudio software (CNIC) for spine number and types (thin, mushroom, or others) with post-hoc manual correction performed blinded to genotype. Neuronal reconstructions of dye-filled neurons were performed using Neurolucida software (MBF Bioscience). For dendritic spine analysis, spine segments selected for imaging were at least 100 µm away from cell body, and 4–6 segments per neuron were sampled. Z-stack images were acquired using a Leica SP8 confocal microscope with a 63x oil lens with a zoom of 3.7, NA 1.3 and step size of 0.2 µm. We included 4–6 neurons/animal and 4 animals/genotype for analysis.

### RNAscope in situ hybridization (ISH)

Brains were flash frozen on dry ice, sectioned at 16 µm on a HM 505 E cryostat (8243-30-1000, Global Medical Instrumentation Inc.), and mounted directly onto microscope slides. RNAscope ISH was conducted according to the manufacturer’s instructions (Advanced Cell Diagnostics; CA). The probes used in this study include: Mm-Camk2ɑ-C1 (Cat. no. 445231; target region 896-1986; Accession number NM_009792.3), Mm-GAD1-C1 (Cat. no. 400951; target region 62-3113; Accession number NM_008077.4) and Mn-Dtnbp1-C2 (Cat. no. 494121-C2; target region 153-1138; Accession number NM_025772.4). Brain sections were imaged on a Leica SP8 confocal microscope. For cell counting, 2 brain sections per animal from a total of 3 animals were analyzed.

### Trace-threat conditioning

Trace threat conditioning was performed as described previously^43,48^. For contextual and cued threat conditioning, the procedures were performed between 1 p.m. and 6 p.m. for 2 days. On day 1, each conditioning session consisted of a 3-min acclimation period followed by five trials of the following structure: a 20 s tone at 90 dB followed by a 20 s trace period and then followed by a mild foot shock (duration 2 s, amplitude 0.7 mA). Inter-trial interval was 4 min. On day 2, mice were returned to the same chamber and context as day 1 for 6 min. The percentage of freezing was calculated using the first 3-min period of the contextual test. For the cued test, day 1 followed the above paradigm; on day 2, the mice were placed in the same chamber that was used on day 1, but with a different context (context B: smooth floor, plexiglass tent, no light and peppermint odor) and acclimated for 3 min followed by 1 trial without shock (a 20 s tone at 90 dB and a 20 s-trace period). The freezing percentage was calculated using the 20 s-trace period for the cued test. All testing was performed using the Near Infrared Fear Conditioning System (Med Associates, Inc.; St. Albans, VT). Freezing behavior was quantified using VideoFreeze software.

### Open field test

The open field test was conducted using Ethovision 8.5 software (Noldus Information Technology, Netherlands). Live tracking was utilized along with center point, nose point, and tail base detection. Mice were placed in a clear plexiglass box (42 cm × 42 cm × 31 cm) with lighting set to 100 lx (center of box). Each session was 30 min.

### Elevated plus maze (EPM)

EPM was conducted using Ethovision 8.5 software (Noldus Information Technology, Netherlands) for live tracking. The lights were adjusted to make both open arms yield a measurement of 30 lx on a light meter. Each trial was scheduled to end after 6 min. At the start of each trial, a mouse was placed in the center of the EPM facing one of the open arms; the initial direction that each mouse faced was counterbalanced as to avoid a bias for either open arm. The amount of time spent in the open arms and closed arms was used for analysis.

### Light/dark box

The light/dark box was conducted using Ethovision 8.5 software for Live tracking. The light/dark box apparatus consisted of one clear bright (200 lx) chamber (28 cm × 28 cm × 31 cm) and a smaller dark (<10 lx) chamber (14 cm × 14 cm × 31 cm) with a small opening. Mice were initially placed in the opening facing the dark chamber. Each trial was run for 5 min.

### Statistical analysis

Biochemical data were analyzed using unpaired Student’s *t*-test and one-way ANOVA followed by Tukey’s post hoc test. Behavioral data were analyzed using unpaired *t*-test and two-way ANOVA with repeated measures (two-way RM ANOVA). Electrophysiological data were analyzed using unpaired Student’s *t*-test and two-way RM ANOVA. Morphological data were analyzed using unpaired Student’s *t*-test and nested ANOVA. Statistical analysis was conducted using Prism 7 (Graphpad). mEPSCs and mIPSCs were analyzed using Mini analysis software (Synaptosoft Inc.). Grubbs’ test (GraphPad Prism) was used to determine significant outliers. Mice were randomized for all tests and recordings.

## Results

### Dysbindin-1 is expressed in the BLA

**Fig. 1:**
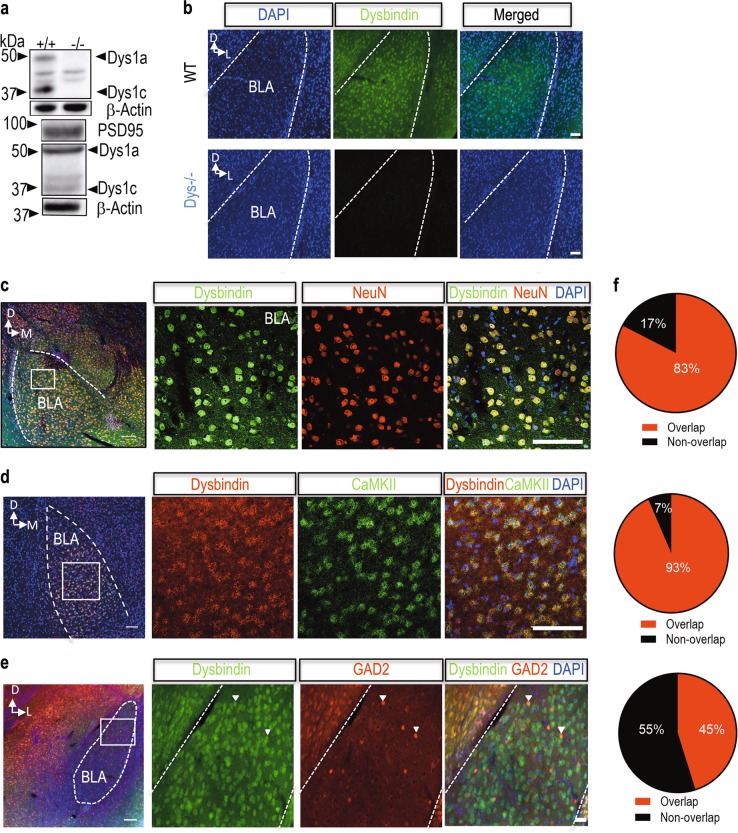
Dysbindin-1 is expressed in excitatory and inhibitory neurons of the amygdala. **a** Top: representative western blots from the amygdala showing that the dysbindin-1 antibody is able to differentiate between Dys1a and Dys1c isoforms in wild-type (WT) mice. These bands are completely eliminated in samples from dysbindin-1 knockout (Dys^−/−^) mice. Bottom: representative western blot images of PSD95 and dysbindin-1 in synaptoneurosome fractions of amygdala from WT mice. **b** Representative images showing dysbindin-1 immunoreactivity in the BLA from a wild-type mouse (upper panels). No such immunoreactivity was detected in a Dys^−/−^ mouse (lower panels) with images taken under the same settings as the WT mouse. **c** Representative image containing the basolateral (BLA) showing dual antigen immunofluorescence for dysbindin-1 (green), NeuN (red; pan-neuronal marker), and DAPI (blue). High-power images of the inset boxes in panel (**c**) showing dysbindin-1 co-localized with NeuN in BLA. **d** Representative image of the BLA showing mRNA expression of both dysbindin-1 (red) and CaMKIIα (green). High-power images from the inset box on the left showing dysbindin mRNA co-localized with the excitatory neuronal marker, CaMKIIα. **e** Representative image of the BLA showing dysbindin-1 protein (green) in the BLA of GAD2-T2a-NLS-mCherry reporter mice. High-power images from the inset box on the left. **f** Pie charts showing the percentage of overlap of dysbindin-1 with other cellular markers (**c–e**). **c**–**e** Scale bar: 100 µm. **b**, **f** Scale bar: 50 µm

The dysbindin-1 protein has three isoforms (dysbindin 1a, 1b and1c). While humans express all three isoforms, mice only express dysbindin 1a and 1c^3^, which we confirmed using brain tissue from wild-type (WT) and Dys^−/−^ mice (Fig. 1a top). Dysbindin-1 has been found in both presynaptic and postsynaptic compartments of hippocampal tissue^3^. We therefore isolated crude synaptoneurosome fractions from the amygdala and found that dysbindin 1a is more concentrated in the synaptic fraction than dysbindin 1c (Fig. 1a bottom). Using dual-antigen immunofluorescence, we investigated what cell types express dysbindin-1 in the amygdala. We first confirmed the specificity of our dysbindin-1 antibody for immunofluorescence using brain tissue from Dys^−/−^ mice (Fig. 1b). Dysbindin-1 protein was expressed only in neurons (neuronal marker NeuN) of the BLA (Fig. 1c), a subregion of amygdala that is necessary for encoding of fear memory and its retrieval^49^. We found that 83% of NeuN^+^ cells expressed dysbindin protein in the BLA (Fig. 1c, f). We then used single molecule fluorescent in situ hybridization (smFISH) to determine the cellular localization of dysbindin-1 mRNA within the amygdala. We found that dysbindin-1 mRNA was expressed in 93% of excitatory neurons (calmodulin kinase II alpha-positive neurons; CaMKIIα mRNA) in the BLA (Fig. 1d, f). We further characterized the expression pattern of dysbindin-1 in inhibitory neurons using GAD2-T2a-NLS-mCherry reporter mice and observed that 45% of GAD2 neurons were colocalized with dysbindin-1 protein in the BLA (Fig. 1e, f). Our results indicate that dysbindin-1 mRNA, as well as protein, is expressed in both excitatory and inhibitory neurons in the BLA.

### Dys^−/−^ mice have impaired threat memory

**Fig. 2:**
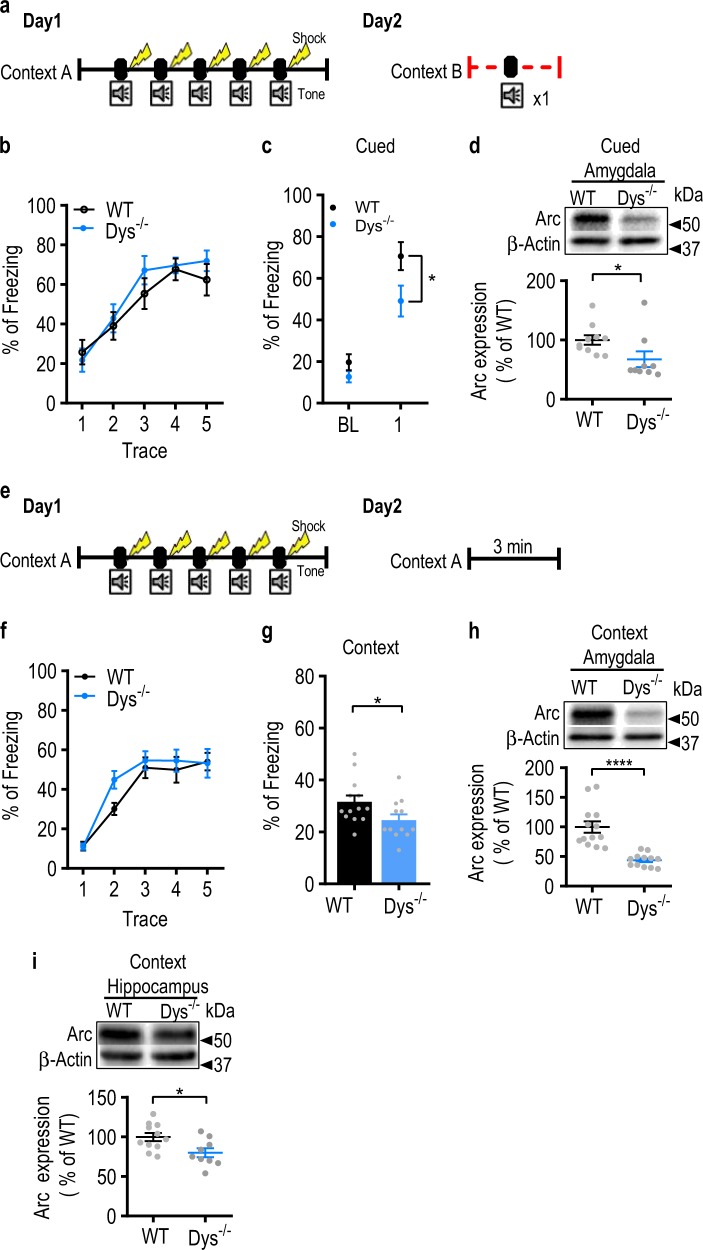
Dys^−/−^ mice display impairments in conditioned threat memory. **a** Schematic illustration of the trace-threat conditioning protocol. **b** Wild-type (WT; *n* = 10 mice; black circles) and dysbindin-1 knockout (Dys^−/−^; *n* = 9 mice; blue circles) mice were subjected to a trace threat-conditioning paradigm on day 1 in context A. The amount of freezing during each of the five trace intervals was measured for each group. **c** Twenty-four hours after threat conditioning, mice were placed in context B and presented a conditioning tone without foot-shock. Freezing was measured during the first 3 min (baseline; BL) and during the 20 s after tone presentation. **d** Arc protein was measured in the amygdala of WT (*n* = 10 mice; black) and Dys^−/−^ (*n* = 9 mice; blue) 30 min after cue retrieval. **e** Schematic illustrating the trace-threat conditioning protocol for context retrieval. **f** WT (*n* = 13 mice; black circles) and Dys^−/−^ (*n* = 12 mice; blue circles) mice were subjected to a trace threat conditioning paradigm on day 1 in context A. The amount of freezing during each of the five trace intervals was measured for each group. **g** Twenty-four hours after conditioning, mice were returned back to context A. The amount of freezing was measured for the first 3 min of the trial. **h** Arc protein was measured in the amygdala of WT (n = 11 mice; black) and Dys^−/−^ (*n* = 9 mice; blue) 30 min after contextual retrieval. (**i**) Arc expression was measured in the hippocampus from WT (*n* = 11 mice; black) and Dys^−/−^ (*n* = 9 mice; blue) mice. Asterisk (*) indicates significant differences from the WT group (*p* < 0.05). All values represent the mean ± SEM

Having shown that dysbindin-1 is expressed in the BLA, we sought to determine whether deletion of dysbindin-1 in mice impairs amygdala-dependent behavior. Pavlovian threat conditioning is a behavioral paradigm that is used to test fear learning and memory, in which the amygdala is critically involved ^49,50^. In auditory threat (fear) conditioning, an auditory cue (conditioned stimulus; CS) is paired with an aversive foot-shock (unconditioned stimulus; US). Re-exposure to the same CS elicits a fear response (i.e. freezing). WT and Dys^−/−^ mice were subjected to a trace threat fear conditioning (five tone-shock pairings; Fig. 2a) on day 1. Twenty-four hours after conditioning, mice were placed in a novel context and presented with the CS (cue retrieval). Both WT and Dys^−/−^ mice conditioned equally well on day 1, as shown by similar levels of freezing (Fig. 2b, genotype: *F*_(1, 17)_ = 0.45, *p* *=* 0.51; two-way RM ANOVA). However, Dys^−/−^ mice froze less during the cue-dependent fear recall (Fig. 2c, *t*_17_ = 2.2, *p* = 0.04; unpaired Student’s *t-*test). The behavioral deficit was associated with reduced protein levels of activity regulated cytoskeletal protein (Arc), a plasticity-associated protein^51,52^, in the amygdala (Fig. 2d, *t*_17_ = 2.1, *p* = 0.04; unpaired Student’s *t*-test). Separate cohorts of mice were subjected to the same procedure on day 1 (Fig. 2e, f, f: genotype: *F*_(1, 23)_ = 1.3, *p* = 0.26; two-way RM ANOVA). On day 2, mice were placed back in the same conditioning chamber to assess contextual memory. Dys^−/−^ mice exhibited lower freezing during the context-dependent recall test (Fig. 2g, *t*_23 = _2.14, *p* = 0.04; Student’s *t* test) on day 2. We also observed lower Arc expression in the amygdala (Fig. 2h, *t*_23_ = 5.4, *p* < 0.0001; unpaired Student’s *t*-test) and in the hippocampus (Fig. 2i, *t*_18_ = 2.6, *p* = 0.017; unpaired Student’s *t*-test) of Dys^−/−^ mice compared to WT mice after contextual testing. Finally, we did not observe any differences between genotypes across several anxiety tests, including the open field test (Supplemental Fig. 1a-b; time in center: *t*_17_ = 0.83, *p* = 0.42; entries into center: *t*_17_ = 1.3, *p* *=* 0.21; unpaired Student’s *t*-test), light-dark box (Supplemental Fig. 1c, *t*_18_ = 0.39, *p* = 0.7; unpaired Student’s *t*-test), and EPM (Supplemental Fig. 1d, open arms: *t*_16_ = 1.7, *p* *=* 0.12; closed arms: *t*_17_ = 1, *p* = 0.32; unpaired Student’s *t*-test). In summary, Dys^−/−^ mice displayed impaired threat memory recall to the tone and context, which was not confounded by differences in baseline anxiety.

### Dendritic spine morphology in the BLA is altered in Dys^−/−^ mice

**Fig. 3:**
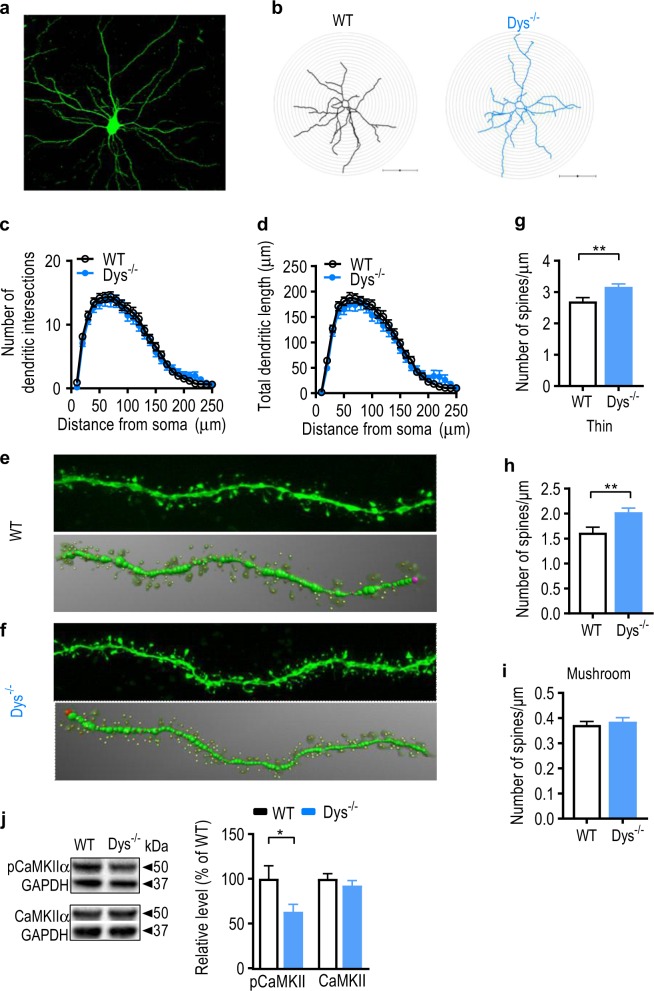
Dendritic spine structure of basolateral amygdala pyramidal neurons is altered in Dys^−/−^ mice. **a** Representative image showing a pyramidal neuron filled with Lucifer yellow in the basolateral amygdala. **b** Representative reconstructions of injected BLA neurons from WT and Dys^−/−^ mice that were used for morphologic analysis. The radius of concentric circles used for Sholl analysis was increased at 10 µm intervals from the soma. Scale bar: 100 µm. Sholl analysis was performed on reconstructed WT (black circles) and Dys^−/−^ (blue circles) mice neurons to analyze dendritic complexity (**c:** intersections; **d:** total dendritic length at each interval). **e** Top: representative confocal image showing a dendritic segment of a filled BLA pyramidal WT neuron used for spine analysis. Bottom: the dendritic segment showed above was traced in 3D using reconstructions obtained from NeuronStudio to analyze spines. Thin spine: yellow color; mushroom spine: brown color. **f** Top: representative confocal image showing a dendritic segment of a filled BLA pyramidal Dys^−/−^ neuron used for spine analysis. Bottom: the dendritic segment showed above was traced in 3D using reconstructions obtained from NeuroStudio to analyze spines. Average **g** total spine density, **h** thin spine density, **i** mushroom spine density was calculated for WT (open bars) and Dys^−/−^ (blue bars) neurons. **g**–**i** For spine analysis, 4–6 dendritic segments per neurons were analyzed. A total of 23 neurons from 4 WT mice and 22 neurons from 4 Dys^−/−^ mice were included for all analyses. **j** Left: representative western blot images showing phosphorylated CaMKIIα (pCaMKIIα) and total CaMKIIα in the amygdala from WT and Dys^−/−^ mice. Right: levels of pCaMKIIα and CaMKIIα were measured in the amygdala from WT (open bars; *n* = 10 mice) and Dys^−/−^ mice (blue bars; *n* = 9 mice). *p < 0.05; **p < 0.01. All values represent the mean ± SEM

As threat memory was diminished and Arc expression was downregulated in Dys^−/−^ mice, we hypothesized that dendritic morphology would be altered in Dys^−/−^ mice because Arc is an essential mediator of activity-dependent synaptic plasticity at the level of dendritic spines, which can undergo structural changes during memory encoding^53,54^. Thus, we explored whether dendritic arborization and spine morphology were perturbed in the BLA of Dys^−/−^ mice. Brains of naïve, WT and Dys^−/−^ mice were fixed and pyramidal neurons in the BLA were ex vivo iontophoretically filled with Lucifer yellow (Fig. 3a) for morphological analyses^55^. Sholl analysis (Fig. 3b) revealed that there was no significant difference between experimental groups in the number of dendritic intersections (Fig. 3c, genotype: *F*_(1, 6)_ = 0.15, *p* = 0.71; intersection: *F*_(24, 191)_ = 0.45, *p* = 0.84; nested ANOVA) or dendritic length (Fig. 3d, genotype: *F*_(1, 6)_ = 0.03, *p* = 0.87; length: *F*_(24, 191)_ = 1.23, *p* = 0.63; nested ANOVA) at increasing distances from the soma. Surprisingly, we found that spine density was increased in Dys^−/−^ mice compared to WT mice (Fig. 3e–g, g: *t*_43_ = 3, *p* = 0.004; unpaired Student’s *t*-test). The elevated spine density was due to an increase specifically in the number of thin spines (Fig. 3h, *t*_43_ = 3, *p* = 0.004; unpaired Student’s *t*-test), but not mushroom spines (Fig. 3i, *t*_43_ = 0.62, *p* = 0.53; unpaired Student’s *t*-test). Furthermore, the amount of phospho-CaMKIIα (pCaMKII α-activated), but not total CaMKIIα, was significantly decreased in the amygdala of Dys^−/−^ mice (Fig. 3j, p-CaMKIIα: *t*_17_ = 2, *p* = 0.04, CaMKIIα: *t*_17_ = 0.91, *p* = 0.37; unpaired Student’s *t*-test). This change in p-CaMKIIα is consistent with the increased number of immature spines, as CaMKIIα is a synaptic molecule required for dendritic spine stabilization^56^.

### Neuronal excitability was decreased, but basal excitatory neurotransmission remained unchanged in the amygdala of Dys^−/−^ mice

**Fig. 4:**
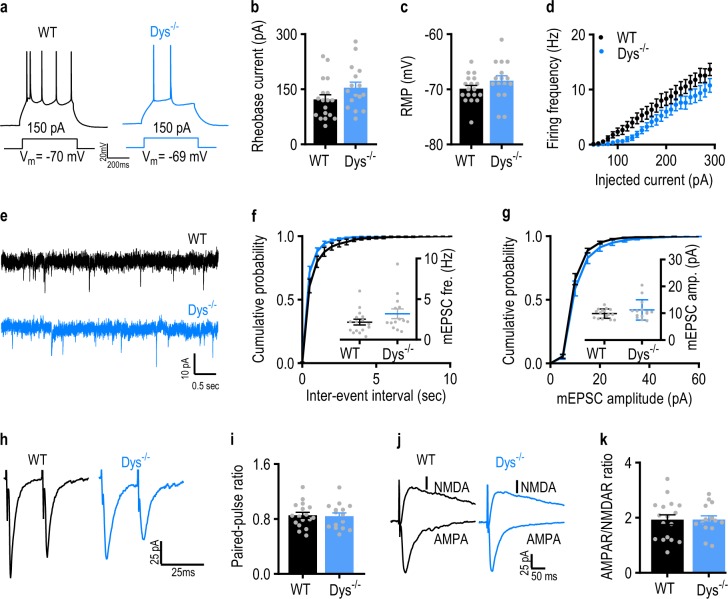
Neuronal excitability is decreased but basal excitatory synaptic transmission is normal in Dys^−/−^ mice. **a** Representative action potential traces recorded from the basolateral amygdala (BLA) of a WT (black) and a Dys^−/−^ mouse (blue) in response to injected current. **b** Bar graphs showing the rheobase current in the BLA of WT (black bar; *n* = 18 cells, six mice) and Dys^−/−^ (blue bar; *n* = 16 cells, six mice) mice. **c** Bar graphs showing the resting membrane potential (RMP) in WT (black bar; *n* = 18 cells, six mice) and Dys^−/−^ (blue bar; *n* = 16 cells, six mice) mice. **d** The evoked firing frequency was lower in the BLA of Dys^−/−^ (blue circles; *n* = 16 cells, six mice) compared to WT (black circles; *n* = 18 cells, six mice) mice. **e** Representative mEPSC traces recorded from the BLA of a WT (black) and Dys^−/−^ mouse (blue). Cumulative probability plots for the distributions of the mEPSC (**f**) inter-event intervals and (**g**) amplitudes from WT (16 cells, six mice) and Dys^−/−^ mice (*n* = 14 cells, six mice). Inset bar graphs summarize the respective average mEPSC (**f**) frequency and (**g**) amplitude. **h** Representative traces of EPSCs evoked by paired presynaptic stimuli with a 50-ms inter-stimulus interval recorded in a WT (left; black) and a Dys^−/−^ mouse (right; blue). **i** Summary plot of paired-pulse ratio measurements for EPSCs recorded in the BLA evoked by thalamic input stimulation in WT (17 cells, four mice) and Dys^−/−^ mice (*n* = 15 cells, 4 mice). **j** Representative traces of AMPA and NMDA receptor-mediated EPSCs recorded in the WT and Dys^−/−^ groups. **k** Bar graph showing the ratio of AMPAR/NMDAR EPSC amplitude ratio in slices from WT (17 cells, four mice; black bars) and Dys^−/−^ mice (*n* = 15 cells, four mice; blue bars). All values represent the mean ± SEM

The deficits in fear expression and dendritic spine abnormalities in Dys^−/−^ mice suggest that neuronal activity and/or synaptic transmission may be dysregulated in the BLA of these mice. To address these possibilities, we first examined the firing patterns of BLA neurons which depend on membrane excitability. We found no differences in the rheobase current, which is a measure of neuronal excitability, or the resting membrane potential between WT and Dys^−/−^ mice (Fig. 4a–c, b: *t*_32_ *=* 1.6, *p* = 0.11; unpaired Student’s *t*-test, c: Mann–Whitney *U* = 100, *p* = 0.12; Mann–Whitney test). However, the frequency of action potentials triggered by prolonged depolarizing current injections in BLA pyramidal neurons under current-clamp recording conditions was lower in slices from Dys^−/−^ mice (Fig. 4a and d, d: stimulation intensity: *F*_(26, 832)_ = 1.13.1, *p* *<* 0.0001; genotype: *F*_(1, 32)_ = 3.52, *p* = 0.07; genotype × stimulation intensity: *F*_(26, 832)_ = 1.79, *p* = 0.0092; two-way RM ANOVA).

The lower firing rates in Dys^−/−^ mice could, at least in part, be due to alterations in excitatory synaptic transmission in the BLA. Therefore, we examined whether dysbindin-1 deficiency affected glutamatergic synaptic transmission in the BLA. We found no differences between control and mutant mice in the frequency (Fig. 4e, f; f: *t*_28_ = 1.5, *p* = 0.13; unpaired Student’s *t*-test) or amplitude (Fig. 4e–g; g: *t*_28_ = 1.3, *p* = 0.19; unpaired Student’s *t*-test) of AMPAR-mediated miniature excitatory postsynaptic currents (mEPSCs). Neither PPR (Fig. 4h, i; i: *t*_30_ = 0.22, *p* = 0.83) nor AMPAR/NMDAR ratio (Fig. 4j, k; k: *t*_30_ = 0.022, *p* = 0.98) were modified in BLA neurons in slices from Dys^−/−^ mice. These results indicate that BLA pyramidal neurons in Dys^−/−^ mice exhibited lower neuronal excitability without changes in excitatory drive at synaptic inputs to these neurons.

### The efficacy of GABAergic inhibition is enhanced in the BLA of Dys^−/−^ mice

**Fig. 5:**
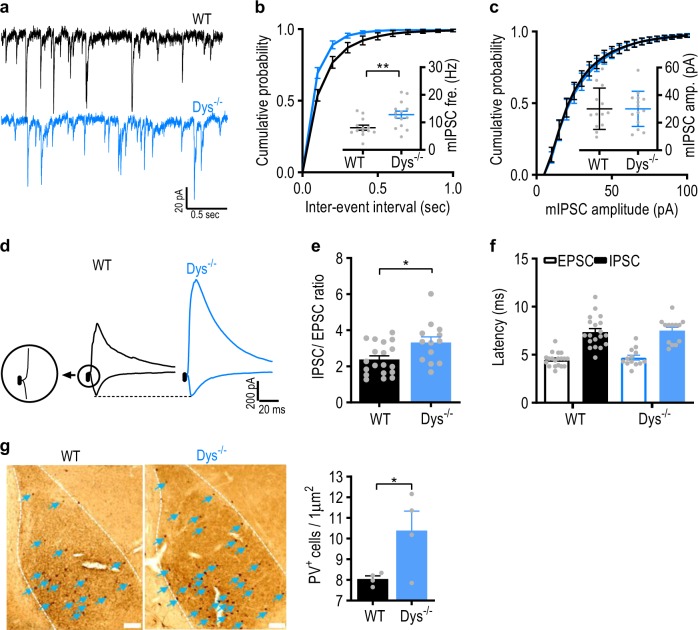
Inhibitory synaptic transmission is enhanced and the number of PV-positive interneurons is increased in the BLA of Dys^−/−^ mice. **a** Representative mIPSC traces recorded in BLA neurons in slices from WT (black) and Dys^−/−^ (blue) mice. **b**, **c** Cumulative probability plots for the distribution of the mIPSC (**b**) inter-event interval and (**c**) amplitude from WT (*n* = 13 cells, 7 mice) and Dys^−/−^ mice (*n* = 14 cells, seven mice). Inset bar graphs summarize the respective mIPSC (**b**) frequency and (**c**) amplitude measurements. **d** Representative traces of EPSCs and IPSCs recorded at −70 or 0 mV, respectively, in the BLA of WT (black) and Dys^−/−^ (blue) mice. The inset circle showing the EPSC had a shorter latency than the IPSC. **e** Bar graph summarizing the IPSC/EPSC ratio values calculated by dividing the amplitude of IPSC by the amplitude of EPSC from WT (*n* = 19 cells, five mice; black bar) and Dys^−/−^ mice (*n* = 13 cells, three mice; blue bar). **f** Summary of the latencies of evoked-EPSCs (open bars) and IPSCs (filled bars) from WT (*n* = 19 cells, five mice) and Dys^−/−^ mice (*n* = 13 cells, three mice). **g** Left: Representative images demonstrating PV-immunoreactive neurons in the BLA from a WT and Dys^−/−^ mouse, respectively. Right: Summary graph showing PV^+^ cells in the BLA of WT (*n* = 4 mice; black bar) and Dys^−/−^ (*n* = 4 mice; blue bar) mice. *p < 0.05. All values represent the mean ± SEM

Since we observed a decrease in neuronal firing, but normal excitatory transmission in Dys^−/−^ mice, we hypothesized that the lower firing rates could at least partially result from an increase in inhibitory drive to BLA neurons. To address this possibility, we assayed GABAergic neurotransmission in the BLA, comparing the parameters of spontaneous and evoked GABA_A_ receptor-mediated synaptic responses between control and Dys^−/−^ mice. We found that the frequency (Fig. 5a, b, b: *t*_25_ = 3, *p* = 0.006; unpaired Student’s *t*-test), but not the amplitude (Fig. 5a and c, c: *t*_25_ = 0.0003, *p* = 0.99; unpaired Student’s *t*-test) of miniature inhibitory postsynaptic currents (mIPSCs) was enhanced in Dys^−/−^ mice compared to WT mice, indicating that BLA neurons in mutant mice may be under tighter tonic inhibitory control compared to WT animals.

We also observed an imbalance of evoked excitatory and inhibitory synaptic transmission in the BLA of mutant mice, as the IPSC/EPSC amplitude ratio was significantly higher in BLA neurons of Dys^−/−^ mice compared to control animals (Fig. 5d, e, e: *t*_30_ = 2.6, *p* = 0.01; unpaired Student’s *t*-test), indicating that the functional efficacy of inhibition at inputs to BLA neurons was enhanced in Dys^−/−^ mice. Under these recording conditions, monosynaptic glutamatergic and disynaptic GABAergic evoked synaptic responses, induced by stimulation of the thalamic input, are recorded from the same neuron^57^. As the IPSC and EPSC in a recorded BLA neuron are induced by presynaptic stimuli of an identical intensity, the IPSC recordings are internally controlled, thus minimizing the variability potentially associated with a position of the stimulation electrode or its physical characteristics. The latencies of both evoked EPSCs and IPSCs were not affected in mutant animals (Fig. 5d and f, f: EPSC: *t*_30_ = 0.69, *p* = 0.49 between genotypes; IPSC: *t*_30_ = 0.27, *p* = 0.79 between genotypes; unpaired Student’s *t*-test).

Notably, we found that the number of parvalbumin immuno-reactive cells (PV+) was increased in the BLA of Dys^−/−^ mice compared to WT mice (Fig. 5g, *t*_6_ = 2.5, *p* = 0.04; unpaired Student’s *t*-test). The increased numbers of PV+ cells in the BLA in mice lacking dysbindin-1 may result in an enhancement of inhibitory drive onto BLA neurons, which in turn could lead to decreases in neuronal activity in the BLA and subsequently impair threat memory consolidation.

## Discussion

Our findings demonstrate that the genetic ablation of dysbindin-1, a protein expressed in both excitatory neurons and inhibitory interneurons of the BLA, impaired the fear response of conditioned threat memory in mice, which was associated with cellular, structural, and functional abnormalities in the amygdala. Specifically, neuronal excitability in the BLA was decreased in Dys^−/−^ mice, and the latter was associated with enhanced GABAergic synaptic transmission, an elevated inhibition/excitation ratio, and an increased number of PV^+^ cells in the BLA of Dys^−/−^ mice. These changes could be translated into reduced neuronal activity in the BLA and subsequently reduced Arc and p-CaMKIIα protein expression in Dys^−/−^ mice. Taken together, our results provide new insights into the functions of dysbindin-1 in the BLA and underscore the importance of dysbinidn-1 contribution to the mechanisms of emotional memory.

We found dysbindin-1 mRNA and protein are expressed in the BLA. Nearly all CaMKIIα-positive cells expressed dysbindin-1. Using several methods to visualize both GABA-synthesizing enzymes (GAD1 and GAD2), we found that dysbindin-1 was located in a sub-population of GABAergic neurons in the BLA (45%). Our results indicate that dysbindin-1 is widely expressed in the BLA. Future studies will investigate whether dysbindin-1 is enriched in particular subpopulations of inhibitory amygdala neurons.

We used a trace-threat-conditioning paradigm, in which the amygdala is critically involved^58–61^, to evaluate the functional significance of dysbindin-1 deletion. In trace-threat conditioning, there is a temporal gap between the CS and US, providing a rapidly acquired model not only for emotional learning and memory, also for working memory and attention-dependent associative learning. Both emotional processing and higher cognitive learning are disrupted in schizophrenia^62,63^. We found that Dys^−/−^ mice displayed impaired threat memory 24 h after conditioning in response to both the CS and conditioned context, which was accompanied by reduced levels of Arc expression in the amygdala and hippocampus. The amygdala is implicated in CS-mediated threat memory formation and retrieval^64,65^. Diminished CS-induced fear responses in Dys^−/−^ mice indicate that dysbindin-1 may play a role in the amygdala gating the mechanisms of threat memory consolidation. While retrieval of contextual threat conditioning is hippocampus-dependent^66^, the observed reduction of activity-dependent Arc expression in the amygdala and hippocampus in contextually conditioned Dys^−/−^ mice is in line with a recent study demonstrating that ventral hippocampal projections to the basal amygdala are necessary for contextual threat memory retrieval^67^. Our results with the context-dependent fear memory test are consistent with previous findings^19^, whereas our data from the cue-dependent fear conditioning differ from prior work^68^. This inconsistency may be due to the differences between mouse strains. Although the amygdala plays a crucial role in threat-associated learning, other brain regions, including the PFC and hippocampus, are also important^49,69^. Thus, future work is needed to evaluate the generality of the observed functional and structural abnormalities, focusing on other components of neural circuits of fear control in addition to the BLA.

The observed impairment of threat memory could be due to disruption in structural and/or functional plasticity. We did not find differences in either the complexity of dendritic arbors or the total amount of dendritic material between genotypes. However, BLA neurons in Dys^−/−^ mice had more dendritic spines than neurons from WT mice. When we classified the spines, we found that the increased number of spines in Dys^−/−^ mice was solely due to an increase in the number of thin spines, but not other types of spines. Thin spines are considered immature and transient with a high turnover rate, while mushroom spines are thought to be mature and stable^70,71^. The hyperactivity of dendritic protrusions could be, at least in part, due to downregulation of CaMKIIα activity in the amygdala of Dys^−/−^ mice. Our findings are consistent with previous reports demonstrating that dysbindin-1 is required for the stabilization of dendritic protrusions through modulating CaMKIIα activity in vitro^15^. Specifically, the deletion of dysbindin-1 increases the proportion of thin spines and filopodia, and also destabilizes dendritic protrusions on cultured hippocampal neurons^15,22^. Since we and others have shown that dysbindin-1 is localized to the postsynaptic compartment, our findings imply that dysbindin-1 may be involved in dendritic stabilization, which may impair neuronal connectivity and integration, similar to that in schizophrenia.

Our ex vivo electrophysiological recordings indicate a hypoexcitability of BLA pyramidal neuronal activity in Dys^−/−^ mice, as BLA neurons in slices from Dys^−/−^ mice had a lower firing frequency in response to depolarizing pulses. Although Dys^−/−^ mice exhibited higher dendritic spine density than WT mice, neither excitatory neurotransmission nor AMPAR/NMDAR ratio were altered in BLA pyramidal neurons in our experiments. This inconsistency between increased spine density without alterations of excitatory neurotransmission in Dys^−/−^ mice, could be due to the increased proportion of thin, highly unstable spines, which do not normally make functional synapses^70^. However, we found that that the frequency of GABA_A_-receptor-mediated spontaneous IPSCs, but not their amplitude, was elevated in Dys^−/−^ mice, which is consistent with the role dysbindin-1 plays in regulating GABA release^9,20^. The inhibition/excitation ratio was enhanced in neurons of Dys^−/−^ mice, indicating that the activity of excitatory neurons could be inhibited in these animals. However, other mechanisms, including changes in ion conductances (e.g., due to altered levels and/or function of voltage-gated Na^+^ and/or K^+^ channels, and/or modifications in slow and fast AHPs and Ih) could potentially also contribute to the observed decreases in neuronal excitability in mutant mice. Specifically, network-level plastic modifications in the BLA, resulting in changes in the frequency of spike-induced sEPSCs, could possibly affect neuronal excitability, thus warranting further investigation. Notably, enhanced GABAergic inhibition has been shown to impair consolidation of threat memory^72^. The increase in inhibitory drive onto the BLA pyramidal neurons in Dys^−/−^ mice could be due, in part, to the observed increase in the number of PV+ cells, providing perisomatic inhibition of principal neurons^73^. Another possibility is that interneurons could become hyperactive through the increased synaptic strength at inputs from PFC. It has been shown that pyramidal neurons in the mPFC project to interneurons within the BLA^45^, and deletion of dysbinidin-1 in the PFC increases the activity of layer 2/3 pyramidal neurons, which in turn elevates neurotransmission onto interneurons in the BLA^20^. The enhanced functional efficacy of inhibition in the BLA in Dys^−/−^ mice could contribute to impairments in the mechanisms of threat memory consolidation and retrieval.

Our finding of enhanced GABAergic transmission in the BLA differs from what has been reported for the mPFC of Dys^−/−^ mice, where the inhibitory drive to pyramidal neurons in layer V of PFC was found to be decreased^74^. The observed differences reinforce the view that the mutation effects may be brain region specific and be translated into region-specific functional modifications (e.g., distinct synaptic and neuronal changes). In the mPFC, impaired GABAergic transmission and fewer inhibitory synapses on pyramidal neurons of Dys^−/−^ mice was apparently due to reduced activity-dependent secretion of brain-derived neurotrophic factor (BDNF) from pyramidal neurons^9,20^. Thus, in the BLA of Dys^−/−^ mice, it is possible that BDNF release is increased from excitatory neurons, thereby enhancing BLA GABAergic transmission. Furthermore, BDNF deletion has been shown to reduce PV expression and cell density^75^. Increased BDNF in the BLA of Dys^−/−^ mice could also explain the increased number of PV neurons, which is in contrast to what has been observed in the dorsolateral PFC of patients with schizophrenia^76^, a region that also exhibits reduced BDNF expression^77^. There are numerous examples in the literature showing that various perturbations can either increase or decrease BDNF expression depending on the brain region^78–81^. Future work will determine whether increased BDNF expression and/or secretion is responsible for the increased inhibitory drive in the BLA of Dys^−/−^ mice.

In summary, we have demonstrated that dysbindin-1 is expressed in both excitatory neurons and inhibitory interneurons in the BLA, an essential part of neural circuits of fear conditioning. In Dys^−/−^ mice, an increase in inhibitory drive to principal neurons in the BLA was associated with a lower firing rate of BLA pyramidal neurons. The resulting reductions in neuronal activity could explain reduced Arc expression and CaMKIIα activation in mutant mice, observed in our studies. As the BLA is critical for fear memory encoding and, possibly, retention, the enhanced inhibitory tone in the BLA could contribute to the impaired threat memory observed in Dys^−/−^ mice. Our findings indicate an important role for dysbindin-1 in regulation of the amygdalar function and suggest potential mechanisms by which dysbindin-1 may contribute to the pathophysiology of schizophrenia.

## Supplementary information

Supplemental figure 1
