# Supplementary material for: Deletion of Dtnbp1 in mice impairs threat memory consolidation and is associated with enhanced inhibitory drive in the amygdala
Source: Transl Psychiatry. 2019 Apr 9;9:132. doi: 10.1038/s41398-019-0465-y (PMC6456574; doi:10.1038/s41398-019-0465-y)
Supplement: Supplementary file 1 — Supplemental figure 1 [file 41398_2019_465_MOESM1_ESM.docx]

**Supplemental Fig. S1 Dysbindin-1 mutant mice do not display changes in anxiety.** (**a-b)** In the open field test, mice were allowed to travel for 30 min. Neither the **a** time spent in the center nor **b** numbers of entries into the center area were different between WT (n=10 mice; black bars) and Dys^-/-^ (n=9 mice; blue bars) mice. **c** In the light/dark box, WT (n=10 mice; black) and Dys^-/-^ (n=10 mice; blue) mice spent a similar amount of time in the lit compartment. **d** In the elevated plus maze, the amount of time spent in the open and closed arms were comparable between WT (n=10 mice; black bars) and Dys^-/-^ (n=9 mice; blue bars) mice.
